# Adjuvant hyperthermic intraperitoneal chemotherapy in patients with colon cancer at high risk of peritoneal metastases: individual patient data meta-analysis

**DOI:** 10.1093/bjs/znaf076

**Published:** 2025-04-29

**Authors:** Julie J M Hamm, Rudolf van den Berg, Eleni-Rosalina Andrinopoulou, E Sophie Zwanenburg, Gijsbert D Musters, Pieter J Tanis, Alvaro Arjona-Sanchez, the COLOPEC and HIPECT4 Collaborator Groups, E S Zwanenburg, C E L Klaver, D D Wisselink, C J A Punt, P Snaebjornsson, J Crezee, A G J Aalbers, A R M Brandt-Kerkhof, A J A Bremers, J W A Burger, H F J Fabry, F T J Ferenschild, S Festen, W M U van Grevenstein, P H J Hemmer, I H J T de Hingh, N F M Kok, M Kusters, L Schoonderwoerd, J B Tuynman, A W H van de Ven, H L van Westreenen, M J Wiezer, D D E Zimmerman, A van Zweeden, M G W Dijkgraaf, G D Musters, M Ortega-Salas Rosa, A Martínez-López, E M Torres-Tordera, B Rufian-Andujar, F Valenzuela-Molina, A Gordon-Suarez, F J Medina-Fernandez, A Gomez-España, F Triviño-Tarradas, M Granados-Rodríguez, M C Vazquez-Borrego, M Garzas-Martin Almagro, I Inmaculada Lasa-Unzué, R Gómez-Sanz, A López-García, M Díez-Alonso, P Hernández-Juara, R Molina-Villaverde, C Castillo Torres, J I Busteros Moraza, J J Segura-Sampedro, R Rafael Morales-Soriano, C Pineño-Flores, A Serrano Del Moral, I Manzanedo, F Pereira, M E Moneva Arce, R Gianchandani-Moorjani, J M Sánchez-González, C Díaz-López, G Hernandez Hernandez, J G Diaz Mejias, M J Hernández Barroso, R M Abreu-Falcon, A Muñoz Hernández, V Castro López Taruella, C Hernandez Pérez, R Afonso, M Viña-Romero, R Perez-Rodriguez, M Heras-Garceau, I Ramos, O Crusellas Maña, M A Lorenzo Liñán, P A Parra Baños, M Carrasco Prats, M Ruiz Marín, E Terol Garaulet, F García Molina, I M Gallarín Salamanca, M González Cordero, A Titos García, S González-Moreno, A Mayol Oltra

**Affiliations:** Department of Surgical Oncology and Gastrointestinal Surgery, Erasmus University Medical Centre, Rotterdam, The Netherlands; Department of Surgery, Erasmus University Medical Centre, Rotterdam, The Netherlands; Department of Biostatistics, Erasmus University Medical Centre, Rotterdam, The Netherlands; Department of Surgery, Amsterdam University Medical Centre, University of Amsterdam, Amsterdam, The Netherlands; Department of Surgery, Zaans Medical Centre, Zaandam, The Netherlands; Department of Surgical Oncology and Gastrointestinal Surgery, Erasmus University Medical Centre, Rotterdam, The Netherlands; Department of Surgery, Amsterdam University Medical Centre, University of Amsterdam, Amsterdam, The Netherlands; Unit of Oncological and Pancreatic Surgery, Reina Sofía University Hospital, Córdoba, Spain; Maimónides Biomedical Research Institute of Córdoba (IMIBIC), Reina Sofia University Hospital, University of Córdoba, Córdoba, Spain

## Abstract

**Background:**

About a quarter of patients with locally advanced colon cancer (pT4) develop locoregional recurrence, including peritoneal metastases. The aim of this individual patient data meta-analysis (IPDMA) was to evaluate the efficacy of adjuvant hyperthermic intraperitoneal chemotherapy (HIPEC) with regard to reducing the locoregional recurrence rate in the overall population and high-risk subgroups of patients with locally advanced colon cancer.

**Methods:**

A systematic literature search was conducted in July 2024 to identify RCTs on adjuvant HIPEC in addition to routine adjuvant systemic chemotherapy in locally advanced colon carcinoma. An IPDMA was performed, with the locoregional recurrence rate as the primary endpoint and disease-free survival (DFS) and overall survival (OS) as secondary endpoints.

**Results:**

The search identified two trials (COLOPEC and HIPECT4). Individual patient data were pooled for 386 patients, of whom 189 patients received adjuvant HIPEC and 197 patients constituted the control group. The median follow-up was 36 (interquartile range 32–60) months. A modified intention-to-treat analysis showed a 36-month locoregional recurrence rate of 16.0% for HIPEC patients and 21.2% for control patients (*P* = 0.295). Predefined subgroup analyses revealed a significant reduction in locoregional recurrence after HIPEC in patients with right-sided tumours (HR 0.56 (95% c.i. 0.48 to 0.67)) (*P* < 0.001). No significant differences in survival were found for the overall study population; low event rates in subgroups did not allow for survival analyses.

**Conclusion:**

Adjuvant HIPEC significantly reduced the locoregional recurrence rate in right-sided locally advanced colon cancer, but not in the overall study population. Definitive conclusions on DFS and OS require longer follow-up.

## Teaser

Adjuvant hyperthermic intraperitoneal chemotherapy (HIPEC) did not significantly improve the locoregional recurrence rate, disease free-survival, and overall survival in patients with locally advanced colon cancer. A significant reduction in locoregional recurrence was found after adjuvant HIPEC in patients with right-sided tumours, especially those with proven pT4 stage tumours. There was a measurable impact of adjuvant HIPEC on intraperitoneal disease recurrence, but this required a specific study population with sufficient a priori risk.

## Introduction

Colorectal cancer (CRC) is the third most common cancer worldwide, with metastatic disease being a leading cause of mortality in patients with CRC^1,2^. The peritoneum is a common site of recurrence, alongside the liver and lungs^3^. Risk factors for the development of peritoneal metastases are a locally advanced tumour (pT4), tumour perforation, mucinous and signet ring cell histology, nodal involvement, a right-sided tumour location, and positive resection margins^4,5^. Peritoneal metastases are associated with a poor prognosis and can cause debilitating symptoms, such as declining performance status, bowel obstruction, malignant ascites, visceral pain, and malnutrition^3,5–10^. This emphasizes the clinical importance of preventing the development of peritoneal metastases in patients with CRC.

Peritoneal metastases are often diagnosed at an advanced stage and therefore only selected patients are considered candidates for surgical treatment^10^. A recent systematic review showed a median overall survival (OS) of 33.6 (range 12–63) months after cytoreductive surgery (CRS) and hyperthermic intraperitoneal chemotherapy (HIPEC)^11^. For most patients, only palliative treatment options remain, offering a considerably shorter median survival of 10–14 months^12^. Without treatment, patients with peritoneal metastases face a prognosis of approximately 5 months^12^.

Because of the poor prognosis and limited curative treatment options, strategies to prevent the development of peritoneal metastases are warranted. Although adjuvant systemic chemotherapy is commonly used in patients with pathological T4 CRC, many patients still develop peritoneal metastases. This is potentially due to the relative resistance of peritoneal metastases to systemic therapy^13,14^. Consequently, other treatment modalities should be further examined. Recent RCTs have compared adjuvant HIPEC in combination with adjuvant systemic chemotherapy with a control arm of adjuvant systemic chemotherapy alone, aiming to prevent the development of peritoneal metastases^15,16^.

Trials conducted so far have been relatively small, providing limited statistical power to explore treatment effects in specific subgroups among the T4 population who are at increased risk. The aim of this study was to evaluate the efficacy of adjuvant HIPEC in combination with adjuvant systemic chemotherapy with regard to reducing the locoregional recurrence rate in the overall population of patients with locally advanced colon cancer and predefined subgroups, in comparison with adjuvant systematic chemotherapy alone.

## Methods

### Identification of studies and collection of data

In accordance with the PRISMA guidelines, a systematic literature search was performed in July 2024 to identify all RCTs reporting on adjuvant HIPEC for CRC^17^. The search was conducted in MEDLINE, Embase, Cochrane, Google Scholar, and the Web of Science Core Collection, without any date restrictions. The following keywords were used: colorectal cancer, hyperthermic intraperitoneal chemotherapy, and randomized controlled trial. All relevant keyword variations were used for these terms (*Table S1*). Exclusion criteria were non-English articles, synchronous metastases, non-curative intent of treatment, and preoperative chemotherapy or radiotherapy for the primary tumour. Additionally, the reference lists of all included trials were assessed to identify additional papers.

Two independent reviewers (J.J.M.H. and R.v.d.B.) screened titles and abstracts of all articles. Studies that met the inclusion criteria were selected for full-text review. Disagreements between both reviewers were solved by discussion and consultation with a third reviewer (P.J.T.).

Individual patient data were retrieved by contacting the principal investigators of all eligible trials providing signed data sharing agreements between institutions. The following baseline and treatment data were collected: sex, age, ASA grade, BMI, localization of the primary tumour, pathological tumour category (pT category), nodal status (pN category), mismatch repair status, histology, surgical management, adjuvant HIPEC, adjuvant systemic chemotherapy, and length of follow-up (months). Collected outcome data were occurrence of any recurrence, with location and time of detection, and survival status at the end of follow-up. All data sets were integrated into a single format after harmonization of the baseline, treatment, and outcome variables. A new variable was added to identify the individual trial. Published baseline characteristics from the original trials were examined and compared with the received data. Risk of bias in the included trials was assessed by two independent investigators (J.J.M.H. and R.v.d.B.), according to the revised Cochrane risk-of-bias tool for randomized trials (RoB 2)^18^. Assessment of publication bias using a funnel plot or Egger’s regression test was not feasible, due to the low number of included trials.

### Outcome parameters

The primary outcome was the locoregional recurrence rate. Locoregional recurrence was defined as recurrence of CRC in the abdominal cavity involving the peritoneal surfaces or the tumour bed. This included peritoneal, omental, and ovarian metastases, local recurrence at the retroperitoneal dissection surface, tumour implants at abdominal scars, and anastomotic recurrence. Nodal mesocolic recurrence and recurrence in non-regional (for example para-aortic) lymph nodes were not included in the primary outcome. Occurrence of locoregional recurrence was assessed using carcinoembryonic antigen (CEA) testing and imaging (CT, MRI, or PET), which was followed by percutaneous biopsy or diagnostic laparoscopy for histological assessment when indicated or as part of the trial protocol (COLOPEC)^19^.

The secondary outcomes were disease-free survival (DFS) and OS. DFS was defined as the time to disease recurrence, death, or the end of follow-up (censored). Disease recurrence was diagnosed using CEA testing, imaging, and cytological or histological assessment. OS was defined as the time to death due to any cause. Patients who were alive at the last follow-up were censored. All outcome measurements were analysed from the time of surgical treatment of the primary tumour up to the event or the end of follow-up.

### Statistical analysis

All analyses were conducted using R software, version 4.2.3 (15 March 2023)^20^. A one-stage individual patient data meta-analysis (IPDMA) was performed on the integrated data set after harmonization of data from the included randomized trials^21^. Descriptive statistics are used to summarize baseline characteristics. Continuous variables were compared using Student’s *t* test for normally distributed data and the Kruskal–Wallis test for non-normally distributed data. Categorical variables were compared using the chi-squared test when normally distributed and Fisher’s exact test when non-normally distributed. A time-to-event analysis for all included patients was conducted on an intention-to-treat (ITT) basis, using a log rank test with a one-sided *P* value. This was altered to a modified ITT analysis, as one of the trials applied exclusion criteria on the initially randomized study population^16^. Trial and centre (nested under trial) were used as clustering terms to compare groups with and without the addition of adjuvant HIPEC. Kaplan–Meier curves were created and a Cox regression analysis was conducted for an explorative subgroup analysis. Clinically relevant subgroups were defined based on criteria that have been associated with the risk of peritoneal dissemination in the literature. These criteria were a right-sided tumour location, pT4, and lymph node metastases (pN1–2). The effect of adjuvant HIPEC on locoregional recurrence in predefined subgroups is reported using absolute percentages, as well as HRs with corresponding two-sided 95% confidence intervals. *P* < 0.050 was considered statistically significant.

## Results

### Literature search

The systematic literature search resulted in a total of 1174 articles. After removal of duplicates, 811 articles were screened by title and abstract. Reference lists of selected articles were checked, which did not result in additional articles of interest. A flow diagram for the study selection process is shown in *Fig. S1*. Two articles met the inclusion criteria, one for the COLOPEC trial^15^ and one for the HIPECT4 trial^16^. Both RCTs assigned patients with locally advanced CRC to an experimental arm (adjuvant HIPEC followed by adjuvant systemic chemotherapy) and a control arm (adjuvant systemic chemotherapy alone).

### Study characteristics and patient populations

The two identified trials were both conducted in Europe, the COLOPEC trial in the Netherlands and the HIPECT4 trial in Spain. The risk of bias was moderate in the COLOPEC trial and high in the HIPECT4 trial. Individual domains of the risk-of-bias tool are described in *Fig. S2*. Detailed study characteristics are provided in *Table S2*. The COLOPEC trial included patients with clinical or pathological T4 N0–2 M0 or perforated CRC from April 2015 to February 2017. Patients assigned to the experimental arm received HIPEC with intravenous fluorouracil (400 mg/m^2^) and leucovorin (20 mg/m^2^), followed by intraperitoneal oxaliplatin (460 mg/m^2^) for 30 min at 42–43°C. HIPEC was delivered simultaneously or within 5–8 weeks after primary tumour resection. The HIPECT4 trial included patients with clinical T4 N0–2 M0 CRC from November 2015 to March 2021. Patients in the experimental arm received HIPEC during primary tumour resection, with mitomycin C (30 mg/m^2^) administered for 60 min at 42–43°C. Patients from both trials were candidates for routine adjuvant systemic chemotherapy consisting of 6 months of capecitabine and oxaliplatin (CAPOX) or fluorouracil and oxaliplatin (FOLFOX).

**Fig. 1:**
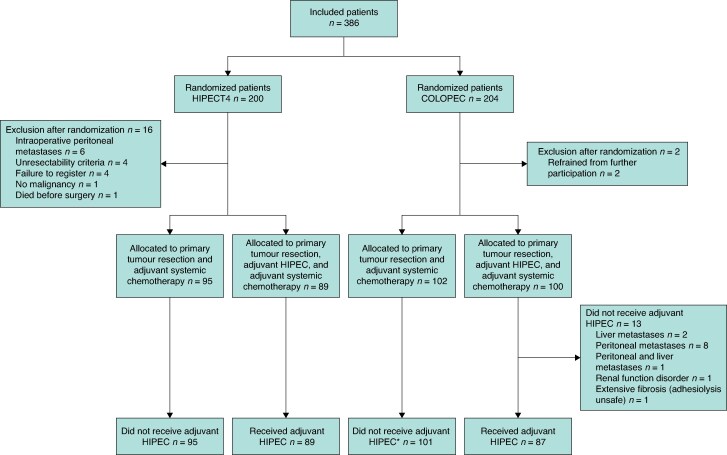
Overview of patient distribution and interventions in the included trials (COLOPEC and HIPECT4) *One patient received adjuvant HIPEC 18 months after surgery. HIPEC, hyperthermic intraperitoneal chemotherapy.

In total, individual patient data were available for 386 patients, of whom 189 were randomized to an experimental treatment with adjuvant HIPEC group and 197 patients constituted the control group. *Figure 1* shows a comprehensive overview of the included patients with corresponding interventions per trial. The COLOPEC trial conducted an ITT analysis, whereas the HIPECT4 trail conducted a modified ITT analysis, due to the exclusion of 16 patients after randomization. The baseline patient and tumour characteristics of the pooled study population are listed in *Table 1*. There were no significant differences between the pooled HIPEC and control group. Baseline characteristics of the study populations of both trials are shown in *Table S3*. Patients in the COLOPEC trial had a higher pT category, a higher pN category, less microsatellite instability, and a different primary tumour histology compared with patients in the HIPECT4 trial. The COLOPEC trial finished after the last included patient reached 5 years of follow-up, resulting in a median follow-up of 59 (interquartile range (i.q.r.) 55–65) months. The follow-up of the HIPECT4 trial was still incomplete at the time of the present analysis, with a median follow-up of 36 (i.q.r. 27–36) months. The median follow-up of the combined trials was 36 (i.q.r. 32–60) months.

**Table 1:** Baseline characteristics

	HIPEC+ (*n* = 189)	HIPEC− (*n* = 197)	*P*
**Sex**			0.574
Male	109 (57.7)	107 (54.3)	
Female	80 (42.3)	90 (45.7)	
Age (years), median (i.q.r.)	61 (55–68)	62 (55–69)	0.337
**ASA grade**			0.179
I	44 (23.4)	60 (30.9)	
II	112 (59.6)	102 (52.6)	
III	30 (16.0)	32 (16.5)	
IV	2 (1.1)	0 (0.0)	
**Obesity**			1.000
BMI <30 kg/m^2^	152 (80.9)	157 (81.3)	
BMI ≥30 kg/m^2^	36 (19.1)	36 (18.7)	
**Localization of the primary tumour**			0.838
Left	109 (57.7)	110 (56.1)	
Right	80 (42.3)	86 (43.9)	
**pT**			0.680
pT1	0 (0.0)	1 (0.5)	
pT2	2 (1.1)	4 (2.1)	
pT3	37 (19.6)	40 (20.5)	
pT4a	107 (56.6)	113 (57.9)	
pT4b	43 (22.8)	37 (19.0)	
**pN**			0.590
pN0	69 (36.7)	81 (41.5)	
pN1	59 (31.4)	54 (27.7)	
pN2	60 (31.9)	60 (30.8)	
**Tumour perforation**			0.665
No	152 (80.4)	162 (82.7)	
Yes	37 (19.6)	34 (17.3)	
**Microsatellite instability**			0.131
No	149 (79.3)	159 (82.0)	
Yes	22 (11.7)	12 (6.2)	
Unknown	17 (9)	23 (11.9)	
**Histology**			0.772
Well-differentiated adenocarcinoma	126 (66.7)	125 (63.8)	
Poorly differentiated/undifferentiated adenocarcinoma	31 (16.4)	30 (15.3)	
Adenocarcinoma with unknown differentiation	3 (1.6)	3 (1.5)	
Mucinous carcinoma	22 (11.6)	27 (13.8)	
Signet ring cell carcinoma	4 (2.1)	9 (4.6)	
Medullary carcinoma/other	3 (1.6)	2 (1.0)	

Values are *n* (%) unless otherwise indicated. HIPEC, adjuvant hyperthermic intraperitoneal chemotherapy; i.q.r., interquartile range.

### Locoregional recurrence, DFS, and OS

**Fig. 2:**
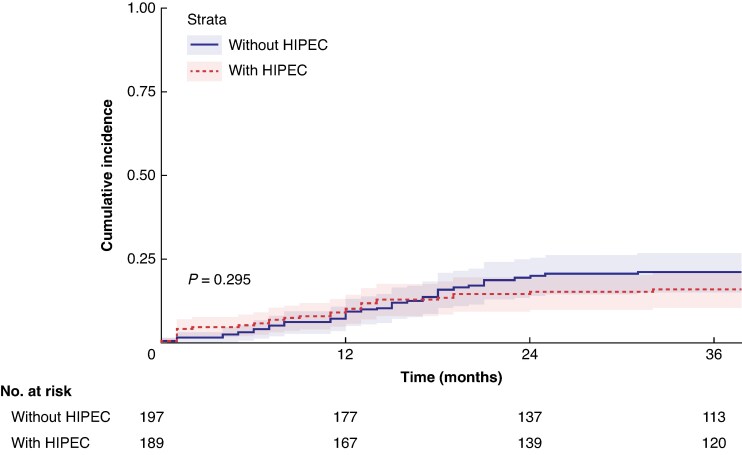
Kaplan–Meier estimates of locoregional recurrence in the overall pooled patient population HIPEC, hyperthermic intraperitoneal chemotherapy.

**Fig. 3:**
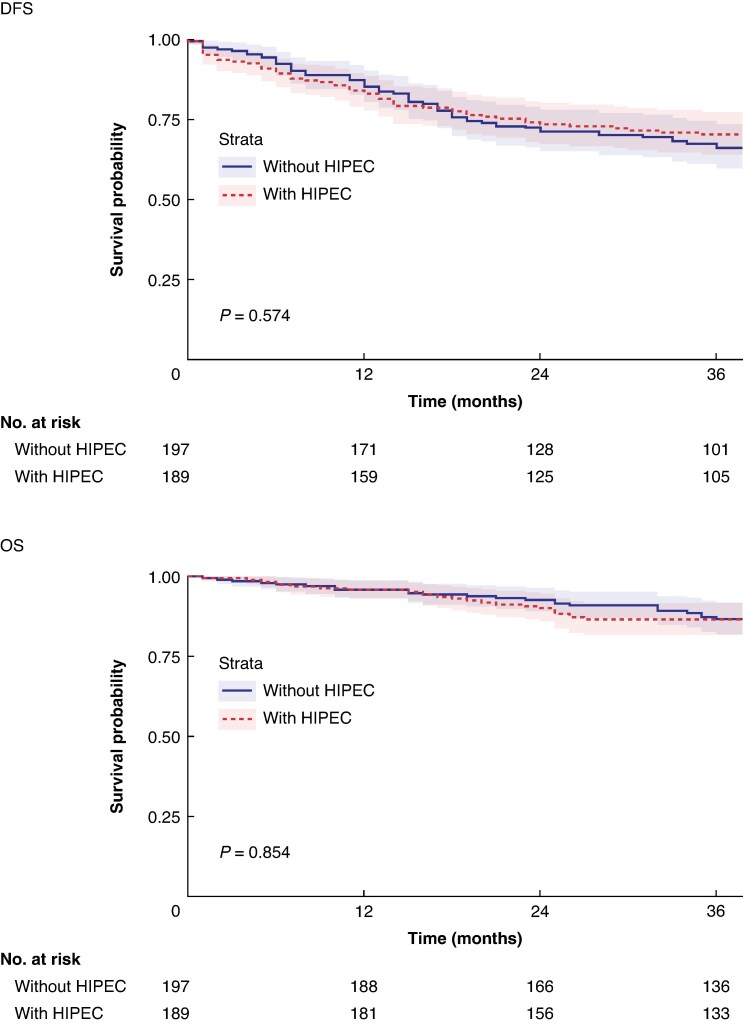
Kaplan–Meier estimates of DFS and OS in the overall pooled patient population DFS, disease-free survival; OS, overall survival; HIPEC, hyperthermic intraperitoneal chemotherapy..

In the pooled data set, locoregional recurrence developed in 29 patients in the HIPEC group and 39 patients in the control group over the complete follow-up interval. The locoregional recurrence rate at 36 months was 16.0% (95% c.i. 10.4% to 21.2%) for patients receiving adjuvant HIPEC and 21.2% (95% c.i. 15.0% to 26.9%) for patients not receiving adjuvant HIPEC; however, this difference did not reach statistical significance (*P* = 0.295) (*Fig. 2*). DFS and OS did not differ between the HIPEC group and the control group (*Fig. 3*). DFS at 36 months was 70.4% (95% c.i. 57.6% to 86.1%) in the HIPEC group and 66.3% (95% c.i. 52.9% to 83.2%) in the control group (*P* = 0.574); the survival curves crossed at 18 months. OS at 36 months was 86.6% (95% c.i. 80.9% to 92.7%) in the HIPEC group and 86.7% (95% c.i. 79.7% to 94.4%) in the control group (*P* = 0.854).

### Predefined subgroup analysis

**Fig. 4:**
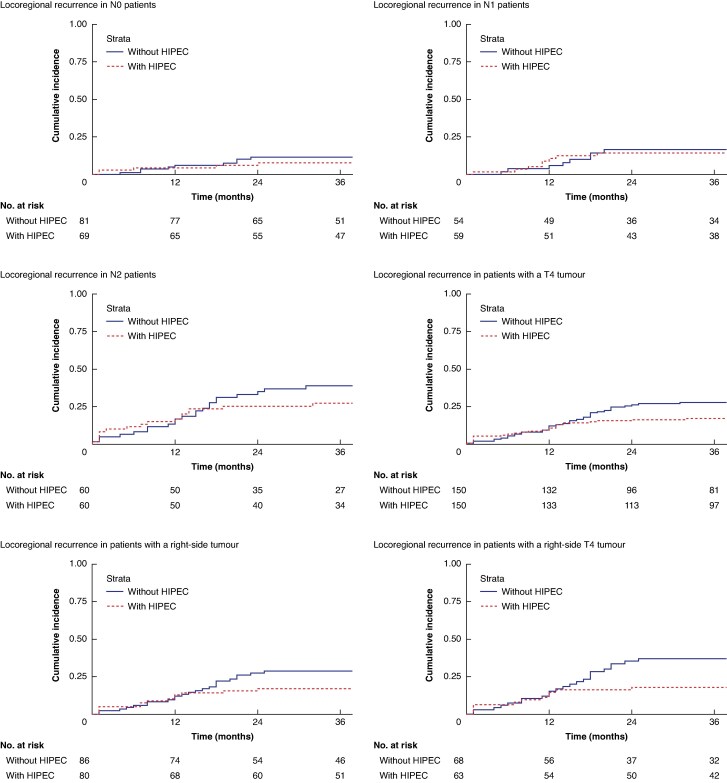
Kaplan–Meier estimates of locoregional recurrence in clinically relevant subgroups based on pT4, pN, and sidedness HIPEC, hyperthermic intraperitoneal chemotherapy.

Kaplan–Meier curves of locoregional recurrence for predefined subgroups are shown in *Fig. 4*. The locoregional recurrence rates at 36 months in the HIPEC arm and the control arm for patients with pT4 were 17% and 28% respectively, with an HR of 0.62 (95% c.i. 0.31 to 1.24) (*P* = 0.173). Corresponding locoregional recurrence rates were 17% and 29% in patients with right-sided tumours, with an HR of 0.56 (95% c.i. 0.48 to 0.67) (*P* < 0.001), 18% and 37% in patients with right-sided pT4, with an HR of 0.47 (95% c.i. 0.29 to 0.77) (*P* = 0.003), and 21% and 29% in patients with pN1–2, with an HR of 0.76 (95% c.i. 0.48 to 1.19) (*P* = 0.228). For left-sided tumours, comparison of locoregional recurrence rates between patients allocated to adjuvant HIPEC and control patients revealed an HR of 1.04 (95% c.i. 0.45 to 2.39) (*P* = 0.931). Due to the low number of events in the pT1–3 subgroup, a Cox regression analysis was not possible. See *Table 2*. The number needed to treat to prevent one locoregional recurrence was 8 for right-sided tumours and 13 for right-sided pT4 tumours.

**Table 2:** Locoregional recurrence in subgroups of patients based on pT4, pN, and tumour location, stratified for HIPEC

Subgroups	Locoregional recurrence (events/total)		HR (95% c.i.)*	*P* *
	HIPEC+	HIPEC−		
pT1–3	4 of 39	1 of 45	**–**	**–**
pT4	25 of 150	39 of 150	0.62 (0.31,1.24)	0.173
Left-sided	16 of 109	16 of 110	1.04 (0.45,2.39)	0.931
Right-sided	13 of 80	24 of 86	0.56 (0.48,0.67)	<0.001
Right-sided and pT4	11 of 63	23 of 68	0.47 (0.29,0.77)	0.003
pN0	5 of 69	10 of 81	0.60 (0.26,1.40)	0.235
pN1–2	24 of 119	30 of 114	0.76 (0.48,1.19)	0.228

^*^Cox regression analysis. HIPEC, adjuvant hyperthermic intraperitoneal chemotherapy.

## Discussion

The result of this IPDMA on adjuvant HIPEC in patients with locally advanced colon carcinoma revealed no significant reduction in the locoregional recurrence rate in the overall study population. Using sidedness as a stratification factor, significantly improved locoregional recurrence rates were found for a right-sided tumour location, with an even lower HR for right-sided tumours with pathologically proven peritoneal penetration (pT4). There was a measurable impact of adjuvant HIPEC on intraperitoneal disease recurrence, but this required a specific study population with sufficient a priori risk. No significant impact on DFS and OS was found, but incomplete follow-up means that definitive conclusions are not possible.

This meta-analysis has several strengths. By using pooled individual patient data, this analysis distinguishes itself from study-level meta-analyses; it provides the best available current evidence for adjuvant HIPEC. All existing patient data from published randomized studies on adjuvant HIPEC were available for this meta-analysis. However, the sample size is still relatively small. Patients were included from numerous hospitals in the Netherlands and Spain, which increases the external validity. Nevertheless, several limitations and methodological issues need to be discussed for better understanding and interpretation of the outcome of this study.

First, there is substantial heterogeneity among the two included trials. The trials differ in primary outcome measure, which was 18-month peritoneal metastases-free survival in the COLOPEC trial and 36-month locoregional control in the HIPECT4 trial. Both outcome measures are unconventional in CRC trials. Furthermore, consensus in the literature on relevant definitions is lacking. Regarding peritoneal metastases, there is controversy concerning the inclusion of recurrent disease at retroperitoneal dissection surfaces, in abdominal wall scars, at the anastomotic site, and in ovaries. Peritoneal metastases are classified as distant disease according to the TNM system, but are often considered regional spread in the abdominal cavity from a pathophysiological and therapeutic perspective (CRS + HIPEC). In the HIPECT4 trial, locoregional recurrence was defined as recurrence in the abdominal cavity involving the peritoneal surfaces or the tumour bed. Detailed data on abdominal recurrence were available from the COLOPEC trial, enabling the composition of the new primary outcome measure, locoregional recurrence. For example, isolated tumour bed recurrence was registered, but not included, in the primary outcome of the COLOPEC trial.

Assessment of the primary outcome measures also differed. In the COLOPEC trail, patients in both arms were assessed using CEA testing and CT, and underwent an additional diagnostic laparoscopy at 18 months if the other modalities did not show signs of recurrence as part of the study protocol. This abdominal exploration identified an additional seven patients with peritoneal metastases in the control group and two in the experimental group, which can be explained by the limited sensitivity of CT in this setting^22,23^. Patients in the HIPECT4 trial did not receive a diagnostic laparoscopy after negative CT, which might have contributed to the overall low proportion of patients with locoregional recurrence in the HIPECT4 trial, especially as the follow-up was incomplete at the time of the present analysis. This low locoregional recurrence rate is also likely to be related to the inclusion of patients based on the presence of clinical T4 (cT4) tumours, resulting in a significantly lower proportion of patients with pT4 (68%) compared with the COLOPEC trial (87%).

Other important differences between the trials are related to the surgical procedure, HIPEC regimens, and the timing of HIPEC. Targeted surgery, including omentectomy, hepatic round ligament resection, appendectomy, and bilateral oophorectomy in post-menopausal females, was performed in the HIPECT4 trial, whereas only resection of the primary tumour was performed in the COLOPEC trial. The added value of the prophylactic resections is unknown. A 60-min mitomycin C (30 mg/m^2^) regimen was used in the HIPECT4 trial, whereas a 30-min bidirectional high-dose oxaliplatin (460 mg/m^2^) regimen was used in the COLOPEC trial. A perfusion time of 60 min, instead of 30 min, allows for increased drug concentrations in tissues and prolonged cytotoxic activity against the residual tumour cells in the peritoneal cavity^24^. However, the duration is still short compared with the generally used 90-min mitomycin C protocol. A 30-min oxaliplatin-based HIPEC regimen has become controversial after the negative outcomes of the PRODIGE 7 trial^25^. Besides the limited perfusion time, concerns are based on the uncertain efficacy with regard to peritoneal metastases after systemic exposure to oxaliplatin, the disadvantage of 5% dextrose as a carrier solution, and the possible adverse effects of hyperthermia^26,27^. There are no randomized studies comparing oxaliplatin and mitomycin C in patients with peritoneal metastases of CRC origin. Regarding the timing of adjuvant HIPEC, staged administration of HIPEC and simultaneous administration of HIPEC each have both benefits and limitations. Most patients in the COLOPEC trial received staged HIPEC (91%), 5–8 weeks after primary surgical resection. Before receiving intended adjuvant HIPEC, nine patients were unexpectedly diagnosed with peritoneal metastases and were treated with CRS + HIPEC if possible. This was possibly caused by missed synchronous peritoneal metastases or rapid disease progression. Because all patients in the HIPECT4 trial received simultaneous HIPEC, this problem did not occur. In contrast, simultaneous HIPEC has the potential for overtreatment, as the indication must be based on clinical parameters, instead of pathological assessment of the tumour. Given the fact that 32% of the patients did not have a pT4 tumour in the HIPECT4 trial after being diagnosed with a cT4 tumour, overtreatment is a substantial threat^28^. On the other hand, adhesions and tumour cell entrapment might interfere with the efficacy of staged HIPEC and this strategy also requires another surgical procedure under general anaesthesia.

The present study indicates some degree of efficacy of adjuvant HIPEC with regard to reducing the locoregional recurrence rate in patients with right-sided colon cancer. Molecular, biological, and embryological differences, depending on tumour sidedness, might have contributed to this observation^29,30^. Similarly, differences in the effectiveness of certain systemic therapies have been observed, depending on tumour sidedness (for example anti-epidermal growth factor receptor (EGFR) agents)^31^. Another explanation might be related to the higher reported risk of peritoneal seeding for right-sided cancers, which makes it easier to detect a statistically significant effect. However, this is not supported by the HR of 1.04 found for left-sided colon cancer.

Future research might focus on improved patient selection (for example sensitive biomarkers) and increasing the effectiveness of adjuvant HIPEC regimens. Alternative intraperitoneal chemotherapy regimens might be explored, such as administration in repetitive cycles, which is one of the basic treatment principles in medical oncology. Better identification of high-risk patients could be a great contribution to intervention studies aiming at prevention of the development of peritoneal metastases and could prevent overtreatment. Pathomics on histological slides of the primary tumour might be a next step in prognostication to identify high-risk patients before surgery, allowing for more tailored adjuvant intraperitoneal chemotherapy.

## Collaborators

**Collaborators COLOPEC:** E. S. Zwanenburg (Amsterdam UMC Location University of Amsterdam, Amsterdam, the Netherlands); C. E. L. Klaver (Amsterdam UMC Location University of Amsterdam, Amsterdam, the Netherlands); D. D. Wisselink (Amsterdam UMC Location University of Amsterdam, Amsterdam, the Netherlands); C. J. A. Punt (UMC Utrecht, Utrecht, the Netherlands); P. Snaebjornsson (Netherlands Cancer Institute, Amsterdam, the Netherlands; Faculty of Medicine, University of Iceland, Reykjavik, Iceland); J. Crezee (Amsterdam UMC Location University of Amsterdam, Amsterdam, the Netherlands); A. G. J. Aalbers (Netherlands Cancer Institute, Amsterdam, the Netherlands); A. R. M. Brandt-Kerkhof (Erasmus Medical Center, Rotterdam, the Netherlands); A. J. A. Bremers (Radboud University Medical Center, Nijmegen, the Netherlands); J. W. A. Burger (Catharina Hospital, Eindhoven, the Netherlands); H. F. J. Fabry (Bravis Hospital, Roosendaal, the Netherlands); F. T. J. Ferenschild (Maashospital Pantein, Beugen, the Netherlands); S. Festen (Onze Lieve Vrouwen Gasthuis, Amsterdam, the Netherlands); W. M. U. van Grevenstein (University Medical Center Utrecht, Utrecht, the Netherlands); P. H. J. Hemmer (University Medical Center Groningen, Groningen, the Netherlands); I. H. J. T. de Hingh (Radboud University Medical Center, Nijmegen, the Netherlands); N. F. M. Kok (Netherlands Cancer Institute, Amsterdam, the Netherlands); M. Kusters (Amsterdam UMC Location University of Amsterdam, Amsterdam, the Netherlands); L. Schoonderwoerd (Bernhoven Hospital, Uden, the Netherlands); J. B. Tuynman (Amsterdam UMC Location Free University, Amsterdam, the Netherlands); A. W. H. van de Ven (Flevo Hospital, Almere, the Netherlands); H. L. van Westreenen (Isala Hospital, Zwolle, the Netherlands); M. J. Wiezer (St Antonius Hospital, Nieuwegein, the Netherlands); D. D. E. Zimmerman (Elisabeth-Tweesteden Hospital, Tilburg, the Netherlands); A. van Zweeden (Amstelland Hospital, Amstelveen, the Netherlands); M. G. W. Dijkgraaf (Amsterdam UMC Location University of Amsterdam, Amsterdam, the Netherlands; Amsterdam Public Health, Amsterdam, the Netherlands); G. D. Musters (Zaans Medical Center, Zaandam, the Netherlands). **Collaborators HIPECT4**: M. Ortega-Salas Rosa (Hospital University Reina Sofia, Cordoba, Spain); A. Martínez-López (Hospital University Reina Sofia, Cordoba, Spain); E. M. Torres-Tordera (Hospital University Reina Sofia, Cordoba, Spain); B. Rufian-Andujar (Hospital University Reina Sofia, Cordoba, Spain); F. Valenzuela-Molina (Hospital University Reina Sofia, Cordoba, Spain); A. Gordon-Suarez (Hospital University Reina Sofia, Cordoba, Spain); F. J. Medina-Fernandez (Hospital University Reina Sofia, Cordoba, Spain); A. Gomez-España (Hospital University Reina Sofia, Cordoba, Spain); F. Triviño-Tarradas (Hospital University Reina Sofia, Cordoba, Spain); M. Granados-Rodríguez (Hospital University Reina Sofia, Cordoba, Spain); M. C. Vazquez-Borrego (Hospital University Reina Sofia, Cordoba, Spain); M. Garzas-Martin Almagro (Hospital University Reina Sofia, Cordoba, Spain); I. Inmaculada Lasa-Unzué (Hospital Príncipe de Asturias, Alcalá de Henares, Madrid, Spain); R. Gómez-Sanz (Hospital Príncipe de Asturias, Alcalá de Henares, Madrid, Spain); A. López-García (Hospital Príncipe de Asturias, Alcalá de Henares, Madrid, Spain); M. Díez-Alonso (Hospital Príncipe de Asturias, Alcalá de Henares, Madrid, Spain); P. Hernández-Juara (Hospital Príncipe de Asturias, Alcalá de Henares, Madrid, Spain); R. Molina-Villaverde (Hospital Príncipe de Asturias, Alcalá de Henares, Madrid, Spain); C. Castillo Torres (Hospital Príncipe de Asturias, Alcalá de Henares, Madrid, Spain); J. I. Busteros Moraza (Hospital Príncipe de Asturias, Alcalá de Henares, Madrid, Spain); J. J. Segura-Sampedro (University Hospital Son Espases, Palma de Mallorca, Spain); R. Rafael Morales-Soriano (University Hospital Son Espases, Palma de Mallorca, Spain); C. Pineño-Flores (University Hospital Son Espases, Palma de Mallorca, Spain); A. Serrano Del Moral (Hospital University of Fuenlabrada, Madrid, Spain); I. Manzanedo (Hospital University of Fuenlabrada, Madrid, Spain); F. Pereira (Hospital University of Fuenlabrada, Madrid, Spain); M. E. Moneva Arce (Hospital University Nuestra Señora de la Candelaria, Tenerife, Spain); R. Gianchandani-Moorjani (Hospital University Nuestra Señora de la Candelaria, Tenerife, Spain); J. M. Sánchez-González (Hospital University Nuestra Señora de la Candelaria, Tenerife, Spain); C. Díaz-López (Hospital University Nuestra Señora de la Candelaria, Tenerife, Spain); G. Hernandez Hernandez (Hospital University Nuestra Señora de la Candelaria, Tenerife, Spain); J. G. Diaz Mejias (Hospital University Nuestra Señora de la Candelaria, Tenerife, Spain); M. J. Hernández Barroso (Hospital University Nuestra Señora de la Candelaria, Tenerife, Spain); R. M. Abreu-Falcon (Hospital University Nuestra Señora de la Candelaria, Tenerife, Spain); A. Muñoz Hernández (Hospital University Nuestra Señora de la Candelaria, Tenerife, Spain); V. Castro López Taruella (Hospital University Nuestra Señora de la Candelaria, Tenerife, Spain); C. Hernandez Pérez (Hospital University Nuestra Señora de la Candelaria, Tenerife, Spain); R. Afonso (Hospital University Nuestra Señora de la Candelaria, Tenerife, Spain); M. Viña-Romero (Hospital University Nuestra Señora de la Candelaria, Tenerife, Spain); R. Perez-Rodriguez (Hospital University Nuestra Señora de la Candelaria, Tenerife, Spain); M. Heras-Garceau (Hospital La Paz, Madrid, Spain); I. Ramos (Hospital de Sant Joan Despí Moises Broggi, Barcelona, Spain); O. Crusellas Maña (Hospital de Sant Joan Despí Moises Broggi, Barcelona, Spain); M. A. Lorenzo Liñán (Hospital de Torrecárdenas, Almería, Spain); P. A. Parra Baños (University General Hospital Reina Sofia, Murcia, Spain); M. Carrasco Prats (University General Hospital Reina Sofia, Murcia, Spain); M. Ruiz Marín (University General Hospital Reina Sofia, Murcia, Spain); E. Terol Garaulet (University General Hospital Reina Sofia, Murcia, Spain); F. García Molina (University General Hospital Reina Sofia, Murcia, Spain); I. M. Gallarín Salamanca (Hospital University Infanta Cristina, Badajoz, Spain); M. González Cordero (Hospital University Infanta Cristina, Badajoz, Spain); A. Titos García (University Regional Hospital, Malaga, Spain); S. González-Moreno (MD Anderson Cancer Center, Madrid, Spain); A. Mayol Oltra (Hospital Provincial Castellón, Castellón, Spain).

## Supplementary Material

znaf076_Supplementary_Data

## Data Availability

Manuscript data are available from the corresponding author upon reasonable request.
