# Supplementary material for: Adjuvant hyperthermic intraperitoneal chemotherapy in patients with colon cancer at high risk of peritoneal metastases: individual patient data meta-analysis
Source: Br J Surg. 2025 Apr 29;112(4):znaf076. doi: 10.1093/bjs/znaf076 (PMC12037274; doi:10.1093/bjs/znaf076)
Supplement: znaf076_Supplementary_Data [file znaf076_supplementary_data.zip › Supplementary_Material.docx]

**Adjuvant hyperthermic intraperitoneal chemotherapy in patients with colon cancer at high risk of peritoneal metastases: An individual patient data meta-analysis**

Julie J. M. Hamm^1^, Rudolf van den Berg^2^, Eleni-Rosalina Andrinopoulou^3^, E. Sophie Zwanenburg^4^, Gijsbert D. Musters^5^, Pieter J. Tanis^1,4^, Alvaro Arjona-Sanchez^6,7^; On behalf of the COLOPEC and HIPECT4 Collaborators groups

^1^Department of Surgical Oncology and Gastrointestinal Surgery, Erasmus University Medical Centre, Rotterdam, the Netherlands.

^2^Department of Surgery, Erasmus University Medical Centre, Rotterdam, The Netherlands.

^3^Department of Biostatistics, Erasmus University Medical Centre, Rotterdam, The Netherlands.

^4^Department of Surgery, Amsterdam University Medical Centre, University of Amsterdam, Amsterdam, The Netherlands.

^5^Department of Surgery, Zaans Medical Centre, Zaandam, The Netherlands.

^6^Unit of Oncologic and Pancreatic Surgery, University Hospital Reina Sofía, Córdoba, Spain

^7^Maimónides Biomedical Research Institute of Córdoba (IMIBIC)/Reina Sofia University Hospital/ University of Córdoba, Spain

**Corresponding author:**

Prof. dr. P.J. Tanis, Professor of Colorectal Surgery

Erasmus Medical Centre, Department of Surgery

Dr. Molewaterplein 40, Rotterdam, the Netherlands

[p.tanis@erasmusmc.nl](mailto:p.tanis@erasmusmc.nl)

**Supplementary Materials - Index**

| **Supplementary Appendixes** |  | |
| --- | --- | --- |
| Collaborators lists | | *page 3* |
| **Supplementary Figures and Tables** | |  |
| Supplementary Figure 1. PRISMA Flow diagram for the study selection process | | *page 8* |
| Supplementary Figure 2. Risk-of-Bias | | *page 9* |
| Supplementary Table 1. Full search terms | | *page 10* |
| Supplementary Table 2. Study characteristics | | *page 11* |
| Supplementary Table 3. Baseline characteristics by trial | | *page 14* |
| **References** | | *page 15* |

**COLOPEC COLLABORATORS GROUP**

E.S. Zwanenburg ^1 2^, C.E.L. Klaver ^1 2^, D.D Wisselink ^1 2^, C.J. A. Punt ^3^, P Snaebjornsson ^4 5^, J. Crezee ^2 6^, A.G.J Aalbers ^7^, A.R.M. Brandt-Kerkhof ^8^, A.J.A. Bremers ^9^, J.W.A. Burger ^10^, H.F.J. Fabry ^11^, F.T.J. Ferenschild ^12^, S. Festen ^13^, W.M.U. van Grevenstein ^14^, P.H.J. Hemmer ^15^, I.H.J.T. de Hingh ^9^, N.F.M. Kok ^7^, M. Kusters ^1 2^, L. Schoonderwoerd ^16^, J.B. Tuynman ^2 17^, A.W.H. van de Ven ^18^, H.L. van Westreenen ^19^, M.J. Wiezer ^20^, D.D.E. Zimmerman ^21^, A. van Zweeden ^22^, M.G.W Dijkgraaf^23 24^, G.D. Musters ^25^

^1^ Amsterdam UMC Location University of Amsterdam, Department of Surgery, Amsterdam, the Netherlands.

^2^ Cancer Center Amsterdam, Treatment and Quality of Life, Amsterdam, the Netherlands.

^3^ UMC Utrecht, Department of Epidemiology, Julius Center for Health Sciences and Primary Care, Utrecht, the Netherlands.

^4^ Netherlands Cancer Institute, Department of Pathology, Amsterdam, the Netherlands.

^5^ Faculty of Medicine, University of Iceland, Reykjavik, Iceland.

^6^ Amsterdam UMC Location University of Amsterdam, Department of Radiation Oncology, Amsterdam, the Netherlands.

^7^ Netherlands Cancer Institute, Department of Surgery, Amsterdam, the Netherlands.

8 Erasmus Medical Center, Department of Oncological and Gastrointestinal Surgery, Rotterdam, the Netherlands.

^9^ Radboud University Medical Center, Department of Surgery, Nijmegen, the Netherlands.

^10^ Catharina Hospital, Department of Surgery, Eindhoven, the Netherlands.

^11^ Bravis Hospital, Department of Surgery, Roosendaal, the Netherlands.

^12^ Maashospital Pantein, Department of Surgery, Beugen, the Netherlands.

^13^ Department of Surgery, Onze Lieve Vrouwen Gasthuis, Amsterdam, the Netherlands.

^14^ University Medical Center Utrecht, Department of Surgery, Utrecht, the Netherlands.

^15^ University Medical Center Groningen, Department of Surgery, Groningen, the Netherlands.

^16^ Bernhoven Hospital, Department of Surgery, Uden, the Netherlands.

^17^ Amsterdam UMC Location Free University, Department of Surgery, Amsterdam, the Netherlands.

^18^ Flevo Hospital, Department of Surgery, Almere, the Netherlands.

^19^ Isala Hospital, Department of Surgery, Zwolle, the Netherlands.

^20^ St Antonius Hospital, Department of Surgery, Nieuwegein, the Netherlands.

^21^ Elisabeth-Tweesteden Hospital, Department of Surgery, Tilburg, the Netherlands.

^22^ Amstelland Hospital, Department of Internal Medicine, Amstelveen, the Netherlands.

^23^ Amsterdam UMC Location University of Amsterdam, Department of Epidemiology and Data Science, Amsterdam, the Netherlands.

^24^ Amsterdam Public Health, Methodology, Amsterdam, the Netherlands

^25^ Department of Surgery, Zaans Medical Center, Zaandam, The Netherlands.

**HIPECT4 COLLABORATORS GROUP**

M. Ortega-Salas Rosa¹, A. Martínez-López¹, E.M. Torres-Tordera², B. Rufian-Andujar², F. Valenzuela-Molina², A. Gordon-Suarez², F.J. Medina-Fernandez², A. Gomez-España³, F. Triviño-Tarradas⁴, M. Granados-Rodríguez⁵, M.C. Vazquez-Borrego⁵, M. Garzas-Martin Almagro⁶, I. Inmaculada Lasa-Unzué⁷, R. Gómez-Sanz⁷, A. López-García⁷, M. Díez-Alonso⁸, P. Hernández-Juara⁸, R. Molina-Villaverde⁹, C. Castillo Torres¹⁰, J.I. Busteros Moraza¹⁰, J.J. Segura-Sampedro^11^, R. Rafael Morales-Soriano¹¹, C. Pineño-Flores¹¹, A. Serrano Del Moral¹², I. Manzanedo¹², F. Pereira¹², M.E. Moneva Arce¹³, R. Gianchandani-Moorjani¹³, J.M. Sánchez-González¹³, C. Díaz-López¹³, G. Hernandez Hernandez¹³, J.G. Diaz Mejias¹³, M.J. Hernández Barroso¹³, R.M. Abreu-Falcon¹³, A. Muñoz Hernández¹⁴, V. Castro López Taruella¹⁵, C. Hernandez Pérez¹⁶, R. Afonso¹⁶, M. Viña-Romero¹⁷, R. Perez-Rodriguez¹⁸, M. Heras-Garceau¹⁹, I. Ramos²⁰, O. Crusellas Maña²⁰, M.A. Lorenzo Liñán²¹, P.A. Parra Baños²², M. Carrasco Prats²², M. Ruiz Marín²², E. Terol Garaulet²², F. García Molina²³, I.M. Gallarín Salamanca²⁴, M. González Cordero²⁴, A. Titos García²⁵, S. González-Moreno²⁶, A. Mayol Oltra²⁷

González-Moreno S. (MD Anderson Cancer Center, Madrid, Spain) Mayol Oltra A. (Unit of Surgery, Hospital Provincial Castellón, Castellón, Spain)

¹ Unit of Pathology, Hospital University Reina Sofia, Cordoba, Spain
² Unit of Surgery, Hospital University Reina Sofia, Cordoba, Spain
³ Unit of Medical Oncology, Hospital University Reina Sofia, Cordoba, Spain
⁴ Unit of Radiology, Hospital University Reina Sofia, Cordoba, Spain
⁵ Maimonides Biomedical Research Institute, IMIBIC, Cordoba, Spain
⁶ Unit of Pharmacy, Hospital University Reina Sofia, Cordoba, Spain
⁷ Unit of Peritoneal Oncologic Surgery, Surgery Department, Hospital Príncipe de Asturias, Alcalá de Henares, Madrid, Spain
⁸ Unit of Coloproctology, Surgery Department, Hospital Príncipe de Asturias, Alcalá de Henares, Madrid, Spain
⁹ Unit of Oncology, Hospital Príncipe de Asturias, Alcalá de Henares, Madrid, Spain
¹⁰ Unit of Pathology, Hospital Príncipe de Asturias, Alcalá de Henares, Madrid, Spain
¹¹ General & Digestive Surgery Department, University Hospital Son Espases; School of Medicine, University of the Balearic Islands; Health Research Institute of the Balearic Islands (IDISBA), Palma de Mallorca, Spain
¹² Surgical Oncology, Hospital University of Fuenlabrada, Madrid, Spain
¹³ Unit of Peritoneal Oncologic Surgery and Colorectal Surgery, Hospital University Nuestra Señora de la Candelaria, Tenerife, Spain
¹⁴ Unit of Radiology, Hospital University Nuestra Señora de la Candelaria, Tenerife, Spain
¹⁵ Unit of Pathology, Hospital University Nuestra Señora de la Candelaria, Tenerife, Spain
¹⁶ Unit of Oncology, Hospital University Nuestra Señora de la Candelaria, Tenerife, Spain
¹⁷ Pharmacy, Hospital University Nuestra Señora de la Candelaria, Tenerife, Spain
¹⁸ Analytics, Hospital University Nuestra Señora de la Candelaria, Tenerife, Spain
¹⁹ Digestive Surgery, Prof. University Autónoma Madrid, Hospital La Paz, Madrid, Spain
²⁰ Unit of Surgery, Consorci Sanitari Integral, Hospital de Sant Joan Despí Moises Broggi, Barcelona, Spain
²¹ Unit of Surgery, Hospital de Torrecárdenas, Almería, Spain
²² Unit of Oncologic Surgery, University General Hospital Reina Sofia, Murcia, Spain
²³ Unit of Pathology, University General Hospital Reina Sofia, Murcia, Spain
²⁴ Unit of Surgery, Hospital University Infanta Cristina, Badajoz, Spain
²⁵ Peritoneal Oncology Surgery Division, Trauma and Emergency Surgery Division, General, Digestive and Transplantation Surgery Department, University Regional Hospital, Malaga, Spain
²⁶ MD Anderson Cancer Center, Madrid, Spain
²⁷ Unit of Surgery, Hospital Provincial Castellón, Castellón, Spain

**Supplementary Figures and Tables**

**Supplementary Figure 1. PRISMA Flow diagram for the study selection process**

Studies included in qualitative synthesis
(n = 2)

Full-text articles assessed for eligibility
(n = 16)

Records excluded
(n = 795)

Records screened
(n = 811)

Records after duplicates removed
(n = 811 )

Additional records identified through other sources
(n = 0)

## Identification

## Eligibility

## Included

## Screening

Records identified through database searching
(n = 1174)

Full-text articles excluded:

Not colorectal origin (n =3)

No randomized controlled trial (n = 2)

Not adjuvant HIPEC (n = 7)

Protocol (n = 4)

Studies included in IPDMA
(n = 2)

HIPEC = hypterthermic intraperitoneal chemotherapy; IPDMA = individual patient data meta-analysis

**Supplementary Figure 2. Risk-of-Bias**


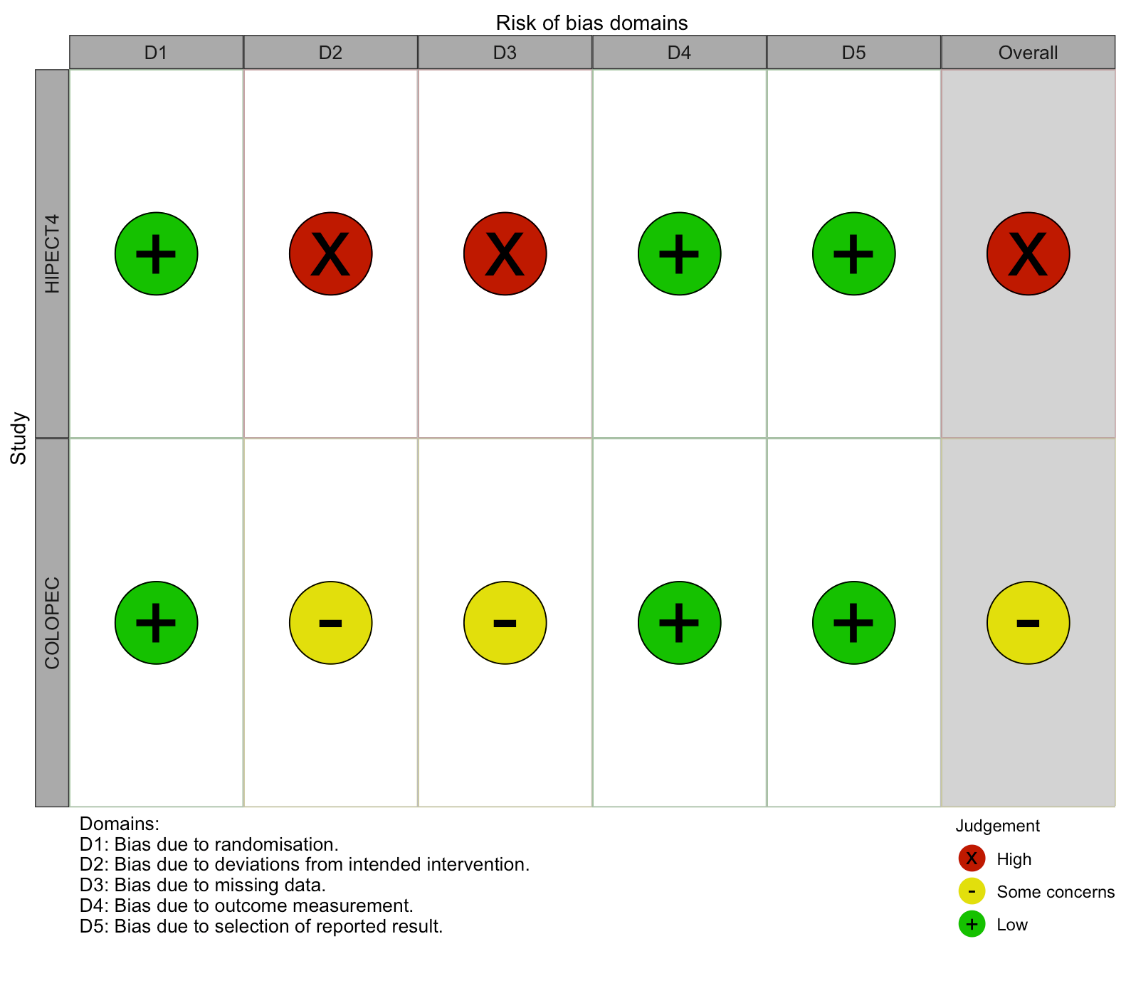


# Supplementary Table 1. Full search terms

| Embase | ('colorectal cancer'/exp OR (((colorect* OR colo* OR rect*) NEAR/3 (cancer* OR tumor* OR tumour* OR neoplas* OR malignan* OR carcinom*))):ab,ti,kw) **AND** ('hyperthermic intraperitoneal chemotherapy'/de OR 'hyperthermic intraperitoneal chemoperfusion'/de OR thermotherapy/de OR ('intraperitoneal drug administration'/de AND (chemotherapy/exp OR 'antineoplastic agent'/exp)) OR (((hypertherm*) NEAR/6 (intraperitoneal OR intra-peritoneal*) NEAR/6 (chemo* OR hypertherm* OR antineoplas* OR anti-neoplas*)) OR HIPEC* OR IPHC OR ((hypertherm*) NEAR/3 (PIC OR chemo*))):ab,ti,kw) **AND** ('randomized controlled trial'/exp OR randomization/exp OR (RCT OR random*):ab,ti,kw) NOT ([Conference Abstract]/lim OR [Conference Review]/lim) NOT ([animals]/lim NOT [humans]/lim) AND [ENGLISH]/lim |
| --- | --- |
| Medline | (exp Colorectal Neoplasms/ OR (((colorect* OR colo* OR rect*) ADJ3 (cancer* OR tumor* OR tumour* OR neoplas* OR malignan* OR carcinom*))).ab,ti,kf.) **AND** (Hyperthermic Intraperitoneal Chemotherapy/ OR ((Peritoneal Lavage/ OR Injections, Intraperitoneal) AND (Chemotherapy, Adjuvant/ OR exp Antineoplastic Agents/)) OR (((hypertherm*) ADJ6 (intraperitoneal OR intra-peritoneal*) ADJ6 (chemo* OR hypertherm* OR antineoplas* OR anti-neoplas*)) OR HIPEC* OR IPHC OR ((hypertherm*) ADJ3 (PIC OR chemo*))).ab,ti,kf.) **AND** (Randomized Controlled Trial/ OR Random Allocation/ OR (RCT OR random*).ab,ti,kf.) NOT (congres* OR abstract*).pt. NOT (exp Animals/ NOT Humans/) AND english.la. |
| Cochrane | ((((colorect* OR colo* OR rect*) NEAR/3 (cancer* OR tumor* OR tumour* OR neoplas* OR malignan* OR carcinom*))):ab,ti) **AND** ((((hypertherm*) NEAR/6 (intraperitoneal OR intra-peritoneal*) NEAR/6 (chemo* OR hypertherm* OR antineoplas* OR anti-neoplas*)) OR HIPEC* OR IPHC OR ((hypertherm*) NEAR/3 (PIC OR chemo*))):ab,ti) NOT "conference abstract":kw |
| Web of Science | TS=(((((colorect* OR colo* OR rect*) NEAR/2 (cancer* OR tumor* OR tumour* OR neoplas* OR malignan* OR carcinom*)))) **AND** ((((hypertherm*) NEAR/5 (intraperitoneal OR intra-peritoneal*) NEAR/5 (chemo* OR hypertherm* OR antineoplas* OR anti-neoplas*)) OR HIPEC* OR IPHC OR ((hypertherm*) NEAR/2 (PIC OR chemo*)))) **AND** ((RCT OR random*)) NOT ((animal* OR rat OR rats OR mouse OR mice OR murine OR dog OR dogs OR canine OR cat OR cats OR feline OR rabbit OR cow OR cows OR bovine OR rodent* OR sheep OR ovine OR pig OR swine OR porcine OR veterinar* OR chick* OR zebrafish* OR baboon* OR nonhuman* OR primate* OR cattle* OR goose OR geese OR duck OR macaque* OR avian* OR bird* OR fish*) NOT (human* OR patient* OR women OR woman OR men OR man))) NOT DT=(Meeting Abstract OR Meeting Summary) AND LA=(English) |
| Google Scholar | "colorectal\|colon\|rectal cancer\|tumor\|tumour\|neoplasm\|malignancy\|carcinoma" "hyperthermic intraperitoneal chemo\|chemotherapy\|chemoperfusion"\|HIPEC\|IPHC RCT\|random\|randomized\|randomly -animal -mouse -mice -rat -rats -conference  'colorectal\|colon\|rectal cancer\|tumor\|tumour\|neoplasm\|malignancy\|carcinoma' 'hyperthermic intraperitoneal chemo\|chemotherapy\|chemoperfusion'\|HIPEC\|IPHC RCT\|random\|randomized\|randomly -animal -mouse -mice -rat -rats -conference |

# Supplementary Table 2. Study Characteristics

|  | **HIPECT4** | **COLOPEC** |
| --- | --- | --- |
| Study design | Multicentre, open-label, randomized clinical trial | Multicentre, open-label, randomized controlled trial |
| Inclusion period | November 15, 2015 – March 9, 2021 | April 1, 2015 – February 20, 2017 |
| Country | Spain | The Netherlands |
| Number of participating centres | 15 | 9 |
| Number of patients included in final analysis | 184 | 202 |
| Inclusion criteria | - Adenocarcinoma of the colon, and colorectal junction which represent cT4a/b in line with The American Joint Committee on Cancer: the 7th edition of the AJCC cancer staging manual; - Male and female patients between the ages of 18 and 75 years; - Lymph node involvement: N0, the presence of N1/2 according to the 7th edition of the AJCC is allowed, provided they can be resectable; - Metastatic extent: M0. - Karnofsky index > 70 or Performance status ≤2. - Informed consent properly completed. | - T4N0-2M0 (T4, either consisting of obvious clinical T4 based on preoperative imaging or intraoperative findings, or pathological T4) - Primary tumor presenting with perforation being curatively resected (N0- 2M0) - Age between 18 and 75 years - Intention to start standard adjuvant systemic therapy - Adequate clinical condition to undergo simultaneous HIPEC or re-laparoscopy   or re-laparotomy with HIPEC within either 10 days or between week 5-8 from primary resection   - Written informed consent - White blood cell count of at least 3000/mm^3^, platelet count of at least 100.000/mm^3^ (< 3 months before surgery) - No bleeding diathesis or coagulopathy - Normal creatinine or creatinine clearance of at least 50 ml/min (< 3 months before surgery) |
| Exclusion criteria | - Presence of metastases (M1) - Incomplete resection or presence of unresectability criteria - Urgent management for obstruction or perforation removing the primary tumor - Extraperitoneal rectal carcinoma; - Preoperative chemotherapy or radiotherapy - Coexistence with other malignant neoplastic disease - Severely altered liver, kidney, or cardiovascular functions - Intolerance to the treatment - Pregnancy or breastfeeding | - Postoperative complications that interfere with adjuvant HIPEC within 8   weeks   - No intention or indication to start standard adjuvant systemic therapy - Non-curative intent treatment - Liver and/or lung metastases - Pathological T4N0 with microsatellite instability (or MMR deficiency based   on immunohistochemistry )   - Pregnant or lactating women - Unstable or uncompensated respiratory or cardiac disease - Serious active infections - Other concurrent chemotherapy - Hypersensitivity to fluorouracil, folinic acid or another substance of leucovorin or oxaliplatin - Stomatitis, ulceration in the mouth or gastrointestinal tract. - Severe diarrhea - Severe hepatic and / or renal dysfunction. - Plasma bilirubin concentrations greater than 85 μmol/l (measurement only   necessary if indicated)   - Pernicious anemia or other anaemias due to vitamin B12 deficiency. - Peripheral sensory neuropathy with functional impairment. |
| Hypothesis | Absolute risk reduction of locoregional recurrence of 18% at 36 months with a significant effect on survival. | Relative risk reduction of peritoneal metastases of 60%, with an absolute difference of 15% at 18-months. |
| Primary Endpoint | Locoregional control rate at 3 years. Locoregional recurrence was defined as relapse of the disease in the abdominal cavity involving the peritoneal surfaces or the tumor bed. | Peritoneal metastasis-free survival at 18 months. Omental and ovarian metastases were considered as peritoneal metastases. |
| Secondary Endpoints | Disease-free survival, overall survival, morbidity, and mortality. | Hospital stay, treatment-related toxicity after HIPEC, disease-free survival, overall survival, quality of life, costs. |
| Sample Size calculation | A locoregional control rate at 36 months of 82% in the experimental arm vs. 64% in the control arm was expected.  To detect an absolute 18% difference in loco-regional control at 36 months a total of 190 patients (95 in each arm) was needed (error ɑ = 0.05, power = 0.80, two-sided), considering a drop-out of 5% the definitive N was 200 patients (100 in each arm). | An absolute 15% difference in peritoneal metastases-free survival at 18 months was expected (18 months peritoneal metastasis-free survival of 90% in the experimental arm vs. 75% in the control arm). A total number of 176 patients (88 patients per arm, with at least 88 assessable patients in the experimental arm) was needed (KaplanMeier, one-sided, alpha=0.05, power of 80%, drop-out 5%). Because some patients had unexpected diagnosis of peritoneal metastases before receiving intended adjuvant HIPEC (and were therefore no longer suitable for adjuvant therapy), the ethics committee approved continuation of randomisation until the predefined number of adjuvant HIPEC procedures was reached. |
| Statistical Analysis | Survival outcomes were compared with a Kaplan-Meier survival analysis with a log-rank test. A modified intention to treat analysis was used, due to exclusion of patients after randomization. | Survival outcomes were compared between the study groups using the Kaplan-Meier survival analysis (log-rank test with one-sided p value), using the intention-to-treat principle. |
| Surgery | All patients received complete cytoreduction of the tumor with targeting surgery by open or laparoscopic approach. Targeting surgery includes: omentectomy, hepatic round ligament, appendicectomy and bilateral oophorectomy in post-menopausian females. | Primary tumor resection, either laparoscopy or open approach. |
| Intervention arm | Intraperitoneal Mitomycin C, 30 mg/m2, diluted in 4 L of dextrose 1.5% solution over 60 minutes. The temperature was required to be between 42 °C and 43 °C during perfusion into the abdominal cavity. | Intraperitoneal oxaliplatin 460 mg/m^2^  (maximal 920 mg) in a single dose, for 30 minutes at a temperature of 42-43 °C. Combined with intraoperative leucovorin 20 mg/m^2^ and 5-fluorouracil 400 mg/m^2^ systemically. |
| Timing of intervention | Simultaneously with resection of the primary tumor. | Simultaneously or 5-8 weeks after resection of the primary tumor. |
| Received intervention | In the investigational group, all 89 patients (100%) received HIPEC with mitomycine C . | In total 87 (87%) of 100 patients in the experimental group received adjuvant HIPEC |
| Systemic adjuvant chemotherapy | Routine adjuvant systemic chemotherapy consisted of  folinic acid, fluorouracil, and oxaliplatin  [FOLFOX] or capecitabine and oxaliplatin[CAPOX]) within 12 weeks of surgery according to local treatment protocols. | Standard adjuvant systemic treatment was given according to the local  institutional protocol, consisting of either capecitabine and oxaliplatin (CAPOX) or 5-FU  and oxaliplatin (FOLFOX). |
| Received systemic adjuvant chemotherapy | In total, 128 patients in the per protocol  population (69.6%) received adjuvant systemic chemotherapy: 63 in the HIPEC group (70.8%) and 65 in the comparator group (68.4%). | The proportion of patients who started adjuvant systemic chemotherapy in each study group was similar (85 [85%] in the experimental group vs 90 [88%] in the control group, p=0·50). |
| Follow-up | Follow-up was performed routinely according to national guidelines during 36 months. Follow-up was performed every 6 months with clinical imaging tests (thorax and abdominal CT mainly or MRI if allergy to intravenous contrast was present) and evaluation of tumor markers (carcinoembryonic antigen and cancer antigen 19-9). | Routine follow-up was performed for at least 5 years. Follow-up included imaging of the liver (ultrasound or CT) at 6 months and 12 months and CT imaging of the abdomen at 18 months, combined with blood carcinoembryonic antigen testing at 3–6 month intervals. If patients developed recurrent disease during this time interval, they were treated accordingly at the discretion of the treating physician. In patients without radiological or pathological diagnosis of recurrent disease at 18 months, or in patients with recurrent disease outside the peritoneal cavity who were still treated with curative intent, a diagnostic laparoscopy was done in both study groups for peritoneal staging. |

# Supplementary Table 3. Baseline Characteristics by trial

|  | **HIPECT4 (n= 184)** | **COLOPEC (n =202)** | p-value |
| --- | --- | --- | --- |
| **Sex, n (%)** |  |  | 0.122 |
| **Female** | 73 (39.7) | 97 (48.0) |  |
| **Male** | 111 (60.3) | 105 (52.0) |  |
| **Age (years), median (i.q.r.)** | 63 (55-69) | 61 (56-68) | 0.187 |
| **ASA- classification, n (%)** |  |  | <0.001 |
| **1** | 22 (12.2) | 82 (40.8) |  |
| **2** | 103 (56.9) | 111 (55.2) |  |
| **3** | 54 (29.8) | 8 (4.0) |  |
| **4** | 2 (1.1) | 0 (0.0) |  |
| **Obesity, n (%)** |  |  | 0.134 |
| **BMI in kg/m^2^ < 30** | 143 (77.7) | 166 (84.3) |  |
| **BMI in kg/m^2^ > 30** | 41 (22.3) | 31 (15.7) |  |
| **Localisation primary tumor , n (%)** |  |  | 0.863 |
| **Left** | 106 (57.6) | 113 (56.2) |  |
| **Right** | 78 (42.4) | 88 (43.8) |  |
| **pT, n (%)** |  |  | <0.001 |
| **pT1** | 1 (0.5) | 0 (0.0) |  |
| **pT2** | 4 (2.2) | 2 (1.0) |  |
| **pT3** | 53 (29.0) | 24 (11.9) |  |
| **pT4a** | 77 (42.1) | 143 (71.1) |  |
| **pT4b** | 48 (26.2) | 32 (15.9) |  |
| **pN, n (%)** |  |  | <0.001 |
| **pN0** | 97 (53.3) | 53 (26.4) |  |
| **pN1** | 43 (23.6) | 70 (34.8) |  |
| **pN2** | 42 (23.1) | 78 (38.8) |  |
| **Perforation, n (%)** |  |  | 0.367 |
| **No** | 154 (83.7) | 160 (79.6) |  |
| **Yes** | 30 (16.3) | 41 (20.4) |  |
| **Microsatellite instability, n (%)** |  |  | <0.001 |
| **No** | 106 (58.9) | 202 (100) |  |
| **Yes** | 34 (18.9) | 0 (0) |  |
| **Unknown** | 40 (22.2) | 0 (0) |  |
| **Histology, n (%)** |  |  | <0.001 |
| **Well differentiated adenocarcinoma** | 104 (56.5) | 147 (73.1) |  |
| **Poorly differentiated/ undifferentiated adenocarcinoma** | 36 (19.6) | 25 (12.4) |  |
| **Unknown differentiation adenocarcinoma** | 32 (17.4) | 17 (8.5) |  |
| **Mucinous carcinoma** | 3 (1.6) | 10 (5.0) |  |
| **Signet-ring cell carcinoma** | 3 (1.6) | 2 (1.0) |  |
| **Medullary/other** | 6 (3.3) | 0(0.0) |  |

# References

1. Bray FA-O, Laversanne M, Sung HA-O, Ferlay J, Siegel RA-O, Soerjomataram I, Jemal A. Global cancer statistics 2022: GLOBOCAN estimates of incidence and mortality worldwide for 36 cancers in 185 countries. CA Cancer J Clin. 2024;74:229-63.

2. Franko J, Shi Q, Meyers JP, Maughan TS, Adams RA, Seymour MT, et al. Prognosis of patients with peritoneal metastatic colorectal cancer given systemic therapy: an analysis of individual patient data from prospective randomised trials from the Analysis and Research in Cancers of the Digestive System (ARCAD) database. Lancet Oncol. 2016;17(12):1709-19.

3. van Gestel YR, de Hingh IH, van Herk-Sukel MP, van Erning FN, Beerepoot LV, Wijsman JH, et al. Patterns of metachronous metastases after curative treatment of colorectal cancer. Cancer Epidemiol. 2014;38(4):448-54.

4. Segelman J, Granath F, Holm T, Machado M, Mahteme H, Martling A. Incidence, prevalence and risk factors for peritoneal carcinomatosis from colorectal cancer. Br J Surg. 2012;99(5):699-705.

5. van Gestel YR, Thomassen I, Lemmens VE, Pruijt JF, van Herk-Sukel MP, Rutten HJ, et al. Metachronous peritoneal carcinomatosis after curative treatment of colorectal cancer. Eur J Surg Oncol. 2014;40(8):963-9.

6. van der Geest LG, Lam-Boer J, Koopman M, Verhoef C, Elferink MA, de Wilt JH. Nationwide trends in incidence, treatment and survival of colorectal cancer patients with synchronous metastases. Clin Exp Metastasis. 2015;32(5):457-65.

7. Aaron J Franke AI, Jason S Starr, Rajesh M Nair, Thomas J George Jr. Management of Malignant Bowel Obstruction Associated With GI Cancers. Journal Oncol Pract. 2017;13(7):426-34.

8. Lemmens VE, Klaver YL, Verwaal VJ, Rutten HJ, Coebergh JW, de Hingh IH. Predictors and survival of synchronous peritoneal carcinomatosis of colorectal origin: a population-based study. Int J Cancer. 2011;128(11):2717-25.

9. Quere P, Facy O, Manfredi S, Jooste V, Faivre J, Lepage C, Bouvier AM. Epidemiology, Management, and Survival of Peritoneal Carcinomatosis from Colorectal Cancer: A Population-Based Study. Dis Colon Rectum. 2015;58(8):743-52.

10. Razenberg L, Lemmens V, Verwaal VJ, Punt CJA, Tanis PJ, Creemers GJ, de Hingh I. Challenging the dogma of colorectal peritoneal metastases as an untreatable condition: Results of a population-based study. European Journal of Cancer. 2016;65:113-20.

11. Parikh MS, Johnson P, Romanes JP, Freitag HE, Spring ME, Garcia-Henriquez N, Monson JRT. Cytoreductive Surgery and Hyperthermic Intraperitoneal Chemotherapy for Colorectal Peritoneal Metastases: A Systematic Review. Dis Colon Rectum. 2022;65(1):16-26.

12. Bakkers C, Lurvink RA-O, Rijken A, Nienhuijs SW, Kok NF, Creemers GJ, et al. Treatment Strategies and Prognosis of Patients With Synchronous or Metachronous Colorectal Peritoneal Metastases: A Population-Based Study. Ann Surg Oncol. 2021;28:9073-83.

13. Franko J, Shi Q, Goldman CD, Pockaj BA, Nelson GD, Goldberg RM, et al. Treatment of Colorectal Peritoneal Carcinomatosis With Systemic Chemotherapy: A Pooled Analysis of North Central Cancer Treatment Group Phase III Trials N9741 and N9841. Journal of Clinical Oncology. 2012;30(3):263-7.

14. Klaver YL, Simkens LH, Lemmens VE, Koopman M, Teerenstra S, Bleichrodt RP, et al. Outcomes of colorectal cancer patients with peritoneal carcinomatosis treated with chemotherapy with and without targeted therapy. Eur J Surg Oncol. 2012;38(7):617-23.

15. Zwanenburg ES, El Klaver C, Wisselink DD, Punt CJA, Snaebjornsson P, Crezee J, et al. Adjuvant Hyperthermic Intraperitoneal Chemotherapy in Patients With Locally Advanced Colon Cancer (COLOPEC): 5-Year Results of a Randomized Multicenter Trial. J Clin Oncol. 2024;42(2):140-5.

16. Arjona-Sanchez A, Espinosa-Redondo E, Gutierrez-Calvo A, Segura-Sampedro JJ, Perez-Viejo E, Concepcion-Martin V, et al. Efficacy and Safety of Intraoperative Hyperthermic Intraperitoneal Chemotherapy for Locally Advanced Colon Cancer: A Phase 3 Randomized Clinical Trial. JAMA Surg. 2023;158(7):683-91.

17. Sterne JAC SJ, Page MJ, Elbers RG, Blencowe NS, Boutron I, Cates CJ, Cheng H-Y, Corbett MS, Eldridge SM, Hernán MA, Hopewell S, Hróbjartsson A, Junqueira DR, Jüni P, Kirkham JJ, Lasserson T, Li T, McAleenan A, Reeves BC, Shepperd S, Shrier I, Stewart LA, Tilling K, White IR, Whiting PF, Higgins JPT. RoB 2: a revised tool for assessing risk of bias in randomised trials. BMJ. 2019;366(l4898).

18. Klaver CEL, Musters GD, Bemelman WA, Punt CJA, Verwaal VJ, Dijkgraaf MGW, et al. Adjuvant hyperthermic intraperitoneal chemotherapy (HIPEC) in patients with colon cancer at high risk of peritoneal carcinomatosis; the COLOPEC randomized multicentre trial. BMC Cancer. 2015;15:428.

19. R Core Team (2023). R: A language and environment for statistical computing. R Foundation for Statistical Computing, Vienna, Austria. URL: https://www.R-project.org/

20. Riley RD, Lambert PC, Abo-Zaid G. Meta-analysis of individual participant data: rationale, conduct, and reporting. BMJ. 2010;340:221.

21. Rivard JD, Temple WJ, McConnell YJ, Sultan H, Mack LA. Preoperative computed tomography does not predict resectability in peritoneal carcinomatosis. The American Journal of Surgery. 2014;207(5):760-5.

22. van ’t Sant I, Engbersen MP, Bhairosing PA, Lambregts DMJ, Beets-Tan RGH, van Driel WJ, et al. Diagnostic performance of imaging for the detection of peritoneal metastases: a meta-analysis. European Radiology. 2020;30(6):3101-12.

23. Cashin P, Sugarbaker PH. Hyperthermic intraperitoneal chemotherapy (HIPEC) for colorectal and appendiceal peritoneal metastases: lessons learned from PRODIGE 7 Review. J gastrointest oncol. 2021;12(Suppl 1):S120-S8.

24. Quenet F, Elias D, Roca L, Goere D, Ghouti L, Pocard M, et al. Cytoreductive surgery plus hyperthermic intraperitoneal chemotherapy versus cytoreductive surgery alone for colorectal peritoneal metastases (PRODIGE 7): a multicentre, randomised, open-label, phase 3 trial. Lancet Oncol. 2021;22(2):256-66.

25. Takemoto M, Kuroda M Fau - Urano M, Urano M Fau - Nishimura Y, Nishimura Y Fau - Kawasaki S, Kawasaki S Fau - Kato H, Kato H Fau - Okumura Y, et al. The effect of various chemotherapeutic agents given with mild hyperthermia on different types of tumours. International Journal of Hyperthermia 2009;19:193-203.

26. Pinto A, Pocard M. Hyperthermic intraperitoneal chemotherapy with cisplatin and mitomycin C for colorectal cancer peritoneal metastases: A systematic review of the literature Review. Pleura Peritoneum. 2019;4(2):20190006.

27. Klaver CE, Gietelink L, Bemelman WA, Wouters MW, Wiggers T, Tollenaar RA, Tanis PJ. Locally Advanced Colon Cancer: Evaluation of Current Clinical Practice and Treatment Outcomes at the Population Level. Journal of the National Comprehensive Cancer Network. 2017 Feb:181-90.

28. Lee GH, Malietzis G, Askari A, Bernardo D, Al-Hassi HO, Clark SK. Is right-sided colon cancer different to left-sided colorectal cancer? - a systematic review. Eur J Surg Oncol. 2015;41:300-8.

29. Lee MS, Menter DG, Kopetz S. Right Versus Left Colon Cancer Biology: Integrating the Consensus Molecular Subtypes. J Natl Compr Canc Netw. 2017;15:1540-1413.

30. Rossini D, Boccaccino A, Carullo M, Antoniotti C, Dima G, Ciracì P, et al. Primary tumour side as a driver for treatment choice in RAS wild-type metastatic colorectal cancer patients: a systematic review and pooled analysis of randomised trials. Eur J Cancer. 2023;184(1879-0852 (Electronic)):106-16.
